# Media composition influences yeast one- and two-hybrid results

**DOI:** 10.1186/1480-9222-13-6

**Published:** 2011-08-15

**Authors:** Ying Liu, Zabeena Merchant, Hao-Ching Hsiao, Kim L Gonzalez, Kathleen S Matthews, Sarah E Bondos

**Affiliations:** 1 Department of Biochemistry and Cell Biology, Rice University, Houston, TX 77005 USA; 2 Department of Molecular and Cellular Medicine, Texas A&M University, College Station, TX, 77843 USA

**Keywords:** Yeast one-hybrid, yeast two-hybrid, protein-protein interaction, accuracy, false positive, false negative

## Abstract

Although yeast two-hybrid experiments are commonly used to identify protein interactions, the frequent occurrence of false negatives and false positives hampers data interpretation. Using both yeast one-hybrid and two-hybrid experiments, we have identified potential sources of these problems: the media preparation protocol and the source of the yeast nitrogen base may not only impact signal range but also effect whether a result appears positive or negative. While altering media preparation may optimize signal differences for individual experiments, media preparation must be reported in detail to replicate studies and accurately compare results from different experiments.

## Findings

The yeast two-hybrid system provides an efficient method for identifying novel protein·protein interactions in small-scale studies and high-throughput screens [1-3]. In this system, the first hybrid is composed of a bait protein fused to a DNA binding protein that recognizes DNA sequences upstream of a *lacZ *reporter gene. The second hybrid protein consists of a strong activation domain fused to a potential protein partner. Interaction between bait and partner proteins localizes the strong activation domain to the *lacZ *promoter, thus activating transcription of the *lacZ *reporter gene. Multiple cycles of *lacZ *transcription, translation, and β-galactosidase cleavage of X-gal generate visible quantities of the blue assay product, 5-bromo-4-chloro-3-hydroxyindole. This sensitive system detects interactions that may be difficult to observe by other means (e.g., co-immunoprecipitation) due to low abundance of the protein complex.

The sensitivity of this method can also lead to several problems: (i) transformation efficiency affects signal strength and is highly dependent on technique [3], (ii) 25% to 50% of detected interactions are estimated to be false positives [2,4,5], and (iii) 55% to 90% of true protein interactions are not observed (false negatives) [2,5]. Furthermore, differences in protein abundance can visibly impact outcome [6,7]. Given these difficulties, it is perhaps not surprising that laboratories using similar assays report different protein interactions [2,5].

Identifying sensitive parameters in yeast two-hybrid experiments could help address these difficulties. Herein, we report both the source of media components and the media preparation protocol can impact yeast one-hybrid and two-hybrid results. In the yeast one-hybrid system, the bait protein, which includes a transcription activation domain, is fused to a DNA binding protein [1]. When the protein of interest is hybridized to a DNA binding domain, this assay can be used to assess the presence and strength of an activation domain [8]. Alternately, the protein of interest can be hybridized to a strong activation domain to screen for DNA binding [9]. The yeast one-hybrid method requires transformation of only one plasmid, resulting in improved transformation efficiency and fewer cellular manipulations [3]. This simpler experimental design reduces experimental variability, allowing focus on systematic issues that arise using this method.

### Experimental Design

Media were prepared according to the protocol in Additional File 1 with varying parameters as discussed below. EGY48-p8op-lacZ yeast were transformed as previously described [4,8,10]. The genome of this reporter host strain (EGY48) includes a wild-type LEU2 gene under the control of a series of LexA operators. The reporter plasmid p8op-lacZ, which carries the lacZ gene under the control of LexA operators, had been stably transformed into this strain [8]. For each DNA tested, cells from a single transformation were divided between plates for each condition to ensure that commonly cited error sources, such as interactions with endogenous proteins, autoactivation, and low transformation efficiencies [1-5], did not contribute to differences in results. The resulting blue color of yeast colonies, which reflects the level of transcription activation, was gauged with a standard color chart (Additional File 2). Analysis by color chart yielded data comparable to analysis by enzyme assay. All experiments were repeated at least three times and yielded consistent results. Yeast nitrogen base sources were Sigma (Y1251-100G), Difco (233520), and Clontech (630421).

### Effects of Yeast Nitrogen Base Source on Yeast-One Hybrid Experiments

We first compared media in which the yeast nitrogen base (YNB) was produced by different companies and the pH was adjusted after sterilization to ensure consistency between media preparations. In general, media made with YNB from Sigma yielded the strongest signal in yeast one-hybrid experiments, whereas media made with Difco YNB yielded intermediate results, and media made with Clontech YNB produced the weakest signal (Table 1 Conditions 1-3). We began experiments using pLexA-Gal4, in which DNA encoding the LexA DNA binding domain is fused to DNA for the Gal4 transcription activation domain. Expression of this hybrid protein should produce a strong positive signal. At only 48 hours post-transformation, yeast colonies transformed with pLexA-Gal4 appeared light blue on media made with Sigma and Difco, whereas colonies on the Clontech media remained white. After 60 hours post-transformation, pLexA-Gal4 colonies on Sigma and Difco media were medium blue, and the colonies on the Clontech media were light blue. Although the results from Difco and Clontech media became indistinguishable after 72 hours, colonies on the Sigma plates were still darker blue. While the source of YNB made a consistent difference, using different lots from the same supplier had no impact on results (Additional File 3).

**Table 1:** Yeast one-hybrid results are strongly dependent on media.

	Yeast Nitrogen Base	pH adjustment^1^	48 hrs observation			60 hrs observation			72 hrs observation		
			
			*Gal4*	*Ubx*	*Pro-2*	*Gal4*	*Ubx*	*Pro-2*	*Gal4*	*Ubx*	*Pro-2*
1	Sigma	+	++	-	-	+++	+	-	++++	++	++
2	Clontech	+	-	-	-	++	-	-	+++	++	++
3	Difco	+	++	-	-	+++	+	-	+++	++	++
4	Sigma	-	+++	-	-	++++	+++	++	++++	+++	++
5	Clontech	-	++	+	-	+++	++	+	+++	++	++
6	Difco	-	-	-	-	++	-	-	++++	+++	-

We examined six media conditions, defined by yeast nitrogen base supplier and pH adjustment post-autoclave.  Difco media can yield false negative data (condition 6) and the other media preparations can yield false positives (conditions 1-5) compared to other transcription activation assay methods [8].  “pH” refers to the pH of the 0.7 M potassium phosphate buffer (KP); “Gal4” stands for pLexA-Gal4, the positive control plasmid expressing the DNA binding domain of LexA fused to the Gal4 transcription activation domain (Clontech).  “Ubx” represents the pLexA-Ubx plasmid, “Pro-2” is the negative control, in which proline mutations disrupt transcription activation by the LexA-Ubx hybrid [8]. The blue shade of colonies is reported here as symbols: “++++” for dark blue, “+++” for medium blue, “++” for light blue, “+” for pale blue, and “–” for white colonies.  See Additional File 2 for color standards. The pH after autoclaving but before addition of KP was 4.67, 5.11, and 4.31 for Sigma, Clontech, and Difco media, respectively. After addition of KP buffer post-autoclaving, the pH of all media was 6.7 ± 0.1.  For conditions 1-3, the pH was adjusted to 7.0.  Therefore pH differences are not the source of the supplier-based differences in results.

To determine whether this media effect is unique to LexA-Gal4, we next tested LexA-Ubx, in which LexA is fused to the *Drosophila *Hox protein Ultrabithorax (Ubx), which activates transcription at a moderate level to yield medium blue colonies [8]. Colonies transformed with pLexA-Ubx yielded a similar trend as pLexA-Gal4: colonies on media containing Sigma and Difco YNB were the first to show blue color (Table 1 Conditions 1-3).

As a negative control, we used LexA-Pro2 in which LexA is fused to a Ubx variant containing mutations A223P/Q224P/T225P/A228P, which disrupt a predicted α-helix required for transcription activation [8]. Importantly, these proline mutants also prevent transcription activation by Ubx in a promoter-reporter assay in *Drosophila *S2 cells, corroborating the yeast results [8]. Whereas we expected colonies expressing LexA-Pro2 to be white, colonies grown on all three types of media were light blue after 72 hours. Consequently, the sensitivity of the signal to changes in media appears to depend on the strength of transcription activation: the strongest activator, LexA-Gal4, generated the largest differences, whereas results for the weakest activator, Pro2, were independent of YNB source for this media preparation method.

Based on our prior results, we expected to see a wide range of color: dark blue (++++) for LexA-Gal4, medium blue (+++) for LexA-Ubx, and white (-) for LexA-Pro2 [8]. However, the results for wild-type and mutant Ubx were similar at 72 hours post-transformation in all three conditions. Furthermore, colonies expressing LexA-Gal4 were lighter than expected when grown on Clontech and Difco media. Thus, the range of signal strengths appears poor for Conditions 1-3 (Table 1).

### Effects of Adjusting Media pH on Yeast-One Hybrid Experiments

Strikingly, simply not readjusting the pH after sterilization resulted in amplification of the signal range. Comparing results for wild-type and mutant Ubx, a slight difference in signal was again observed in Sigma media which persisted to 72 hours (Table 1, Condition 4). However, using Difco YNB without pH re-adjustment not only increased the signal range at 72 hours but also matched prior results in yeast and *Drosophila *cell culture [8]. Specifically, for Condition 3 (Difco, no pH adjustment), the data ranged from light blue (++, Ubx and Pro-2) to medium blue (+++, Gal4), whereas for Condition 6 (Difco, pH adjustment), the data ranged from white (-, Pro-2) to dark blue (++++, Gal4).

Even without readjusting the pH, media using YNB sources did not replicate previous data in yeast and *Drosophila *cell culture. Consequently, media choice can influence the prevalence of false positives and false negatives. Expression of the LexA-Gal4 positive control correctly yielded a strong positive signal at 48 hours on Sigma media, but a false negative signal on Difco media. Likewise, although LexA-Ubx expression rapidly generates a positive signal on Sigma plates, expression of LexA-Ubx on Difco plates gives a false negative response at 60 hours post-transformation, although a positive signal eventually develops after longer incubations. Consequently, the researcher could miss this signal, since the pLexA-Gal4 positive control produces signal at 60 hours. Conversely, the Ubx mutant Pro-2, which should *not *activate transcription, yields a false positive signal on Sigma media at after 60 hours incubation. Therefore, by not re-adjusting the pH after autoclaving, we observed a greater overall range of signal, greater media-dependent differences in signal, and greater differences in signal between transcription activators of different strengths.

### Media Effects on Yeast-Two Hybrid Experiments

Given that the source of the YNB had a profound impact on the results observed in a yeast one-hybrid assay, we reasoned that differences in media might partially account for the variations in data originating from different laboratories in yeast two-hybrid experiments [2,3]. Testing this possibility, we examined the impact of the media source in yeast two-hybrid experiments using a set of control plasmids, in which the T-Antigen-B42 activator chimera should bind a LexA-p53 chimera but not a LexA-Lamin chimera. Similar to yeast one-hybrid experiments, we found the results of yeast two-hybrid data were also dependent on media (Table 2). At five days after transformation, only media produced with Clontech YNB yielded a positive signal, and at six days after transformation, colonies on Clontech media had the strongest signal and Sigma media consistently yielded the weakest signal. Surprisingly, this trend is opposite that observed for yeast one-hybrid experiments, suggesting either the two experimental methods are differentially sensitive, or the magnitude of the media effect is influenced by the proteins assayed.

**Table 2:** The source of yeast nitrogen base also impacts yeast two-hybrid results.

	Yeast Nitrogen Base	pH adjustment	5 days observation		6 days observation	
			
			*p53*	*LAM*	*p53*	*LAM*
1	Sigma	-	-	-	+	-
2	Clontech	-	+	-	+++	-
3	Difco	-	-	-	++	-

In this assay using control plasmids provided by Clontech, pB42AD-T-Antigen should bind pLexA-53, in which the p53 protein is fused to LexA, resulting in blue colonies [12]. Conversely, T-Antigen should not bind Lamin produced by pLexA-LAM [11].

In summary, we have demonstrated that i) media components and preparation methods influence yeast one- and two-hybrid experiments, ii) for yeast one-hybrid experiments, this media sensitivity scales with increasing activation domain strength, and iii) media can determine the range of signal observed in yeast *lacZ *reporter assays. Such differences are sufficient to change data interpretation: experiments that should yield a negative result may appear positive, or vice versa. We observed media-dependent effects in four systems: yeast one-hybrid experiments using either the Gal4 activation domain, wild-type Ubx, or a Ubx mutant lacking activity, and finally yeast two-hybrid experiments reporting on the p53·T-antigen interaction. Therefore, this media sensitivity occurs across a wide range of yeast experiments and may contribute to differences in yeast two-hybrid results observed previously [2,11]. Our results for a single yeast nitrogen base source are consistent even when testing different lot numbers or chemicals differing in age by a decade, suggesting that the differences we observe when the source of the yeast nitrogen base is changed are significant. Since each manufacturer reports the same yeast nitrogen base composition, these differences may be attributed to differences in contaminants of these media components (Table 1). These experiments suggest that careful reporting of chemical sources and preparation methods for yeast media is essential to enable replication of experiments and comparison of data between studies. Finally, these media-dependent differences in results could be exploited to enhance and thus verify weak differences in signals from yeast assays.

## Competing interests Statement

The authors declare that they have no competing interests.

## Authors' contributions

YL discovered the media effect on yeast one- and two-hybrid results. ZM, HCH, and KG collected the remaining data and edited the text. KM and SB wrote and edited the text. All authors have read and approved the final manuscript.

## Supplementary Material

Additional file 1 **Detailed protocol for yeast media preparation**. Click here for file

Additional file 2 **Color scale for evaluating yeast colony color, and color scale data compared with data assaying the activity of the β-galactosidase reporter gene product**. Click here for file

Additional file 3 **Different lots of yeast nitrogen base from the same supplier yield consistent results**. Click here for file
