# Supplementary material for: Media composition influences yeast one- and two-hybrid results
Source: Biol Proced Online. 2011 Aug 15;13:6. doi: 10.1186/1480-9222-13-6 (PMC3177868; doi:10.1186/1480-9222-13-6)
Supplement: Additional file 1 — Detailed protocol for yeast media preparation. [file 1480-9222-13-6-S1.PDF]

Protocol: Preparing yeast media

KP Buffer:

1. Dissolving 17.4 g of K_2HPO_4 (Sigma, P9666) to a final volume of 100 ml (1 M K_2HPO_4).
2. Make 1 M KH_2PO_4 by dissolving 13.6 g of KH_2PO_4 (Sigma, P9791) to a final volume of 100 ml.
3. Use a heated stir plate to dissolve both salts. Do not heat above 50 degrees. Do not heat after all of the salt is in solution.
4. Mix 61.5 ml of 1 M K_2HPO_4 with 28.5 ml KH_2PO_4.
5. Adjust the pH to 7.0 if necessary using the appropriate 1 M salt solutions.
6. Dilute the resulting 1 M solution at pH=7.0 to 0.7 M.
7. Filter sterilize the resulting buffer using a 0.20 or 0.22 micrometer cutoff filter into an autoclaved flask or bottle.
8. This "KP buffer" should be stored at 4 ºC and used within one month. Discard if visibly contaminated.

Yeast media plates:

The procedures for making different yeast media plates are similar, but the identity of the dropout supplements, sugars, and antibiotic can vary. Since we have used the Clontech pLexA Matchmaker yeast two hybrid system, we must replace glucose with galactose and raffinose to induce chimera expression.

By way of example, the instructions for making Gal/Raf/-His/-Ura/-Trp/X-gal/Kanamycin plates are described below.

1. Add 300 ml water to a 1 L flask.
2. Using a stir plate, stir continuously without heat while adding:
    a. 0.85 g Yeast nitrogen base without amino acids or ammonium sulfate (Difco #233520). The impact of using yeast nitrogen base from other companies is discussed in the accompanying paper.
    b. 2.5 g Ammonium sulfate (Mallinkrodt Chemicals, 7725)
    c. 10 g Galactose (Acros, 150611000)
    d. 5 g Raffinose (Acros, 1956171000)
    e. 0.375 g –His/-Ura/-Trp dropout supplement (Clontech, 630424)
3. Bring the final volume to 445 ml.
4. Add 10.5 g agar (Fisher Scientific, BP1423). The new volume should be approximately 450 ml.
5. Autoclave at a setting of 1.27 kg/cm^2 at 121 ºC for 15-30 minutes. Clontech recommends 15 minutes, but we use 30 minutes without adverse effects.
6. Allow the flask to cool down. It is sufficiently cool when you can comfortably touch the bottom of the flask – where the liquid is located – to the inside of your arm (preferred method), or you can hold the flask in your hands for 30 seconds. If you proceed to step 7 too quickly, you will destroy the heat-sensitive reagents.
7. Using a sterile hood, add the following reagents while swirling the flask to prevent local cooling / solidification of the agar:

a. 500 μl of 50 mg/ml kanamycin monosulfate (CRPI, K22000) for a final concentration of 50 μg/ml
b. TURN off hood and room lights to add 0.4 ml of 50 mg/ml X-gal (Progene V3941) to a final concentration of 40 μg/ml. X-gal is light sensitive. If you make your own X-gal solution, use DMSO or DMF as a solvent. Either way, your X-gal solution should be clear. If you see traces of brown, it should be discarded.
c. 50 ml 0.7M KP buffer.
