# Supplementary material for: Media composition influences yeast one- and two-hybrid results
Source: Biol Proced Online. 2011 Aug 15;13:6. doi: 10.1186/1480-9222-13-6 (PMC3177868; doi:10.1186/1480-9222-13-6)

Additional File 2.

Color scale for evaluating yeast colony color.

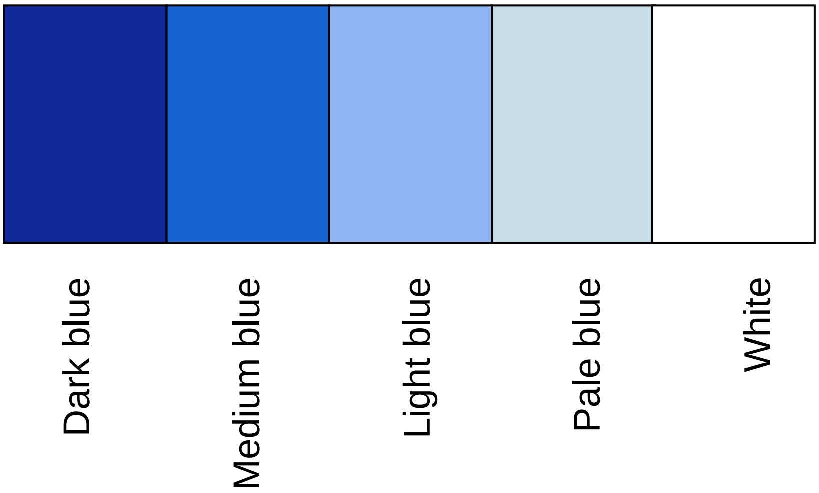

Comparison of colony color, recorded by the above color scale in Table 2, with quantitative results from the β-galactosidase liquid assay, reported in Miller units. Bars are shaded to match the corresponding colony color.

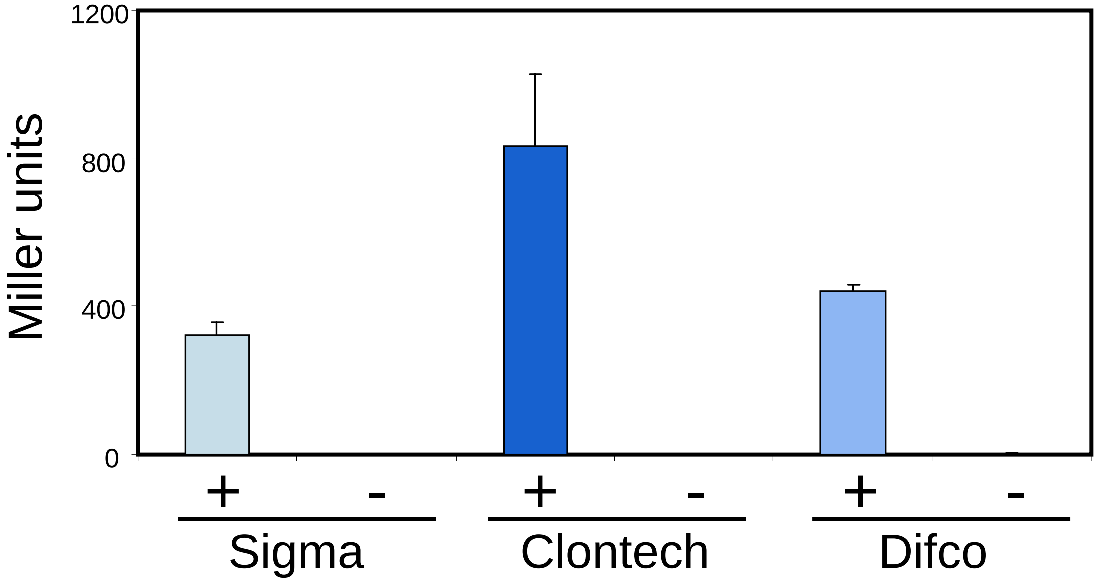

Supplement: Additional file 2 — Color scale for evaluating yeast colony color, and color scale data compared with data assaying the activity of the β-galactosidase reporter gene product. [file 1480-9222-13-6-S2.PDF]
