# Supplementary material for: Media composition influences yeast one- and two-hybrid results
Source: Biol Proced Online. 2011 Aug 15;13:6. doi: 10.1186/1480-9222-13-6 (PMC3177868; doi:10.1186/1480-9222-13-6)
Supplement: Additional file 3 — Different lots of yeast nitrogen base from the same supplier yield consistent results. [file 1480-9222-13-6-S3.PDF]

Additional File 3

Assay results are consistent between different lots from the same supplier. Results for Difco media are shown.

Difco Lot	Expiration Date	48 hrs observation			60 hrs observation			72 hours observation		
		Gal 4	*Ubx*	*Pro-2*	*Gal4*	*Ubx*	*Pro-2*	*Gal4*	*Ubx*	*Pro-2*
144069	12-31-2004	-	-	-	++	-	-	++++	+++	-
7116446	10-30-2012	-	-	-	++	-	-	++++	+++	-
8190329	11-30-2012	-	-	-	++	-	-	++++	+++	-
9194329	6-30-2014	-	-	-	++	-	-	++++	+++	-
